# Oxidative Stress in Ozone-Induced Chronic Lung Inflammation and Emphysema: A Facet of Chronic Obstructive Pulmonary Disease

**DOI:** 10.3389/fimmu.2020.01957

**Published:** 2020-09-02

**Authors:** Coen H. Wiegman, Feng Li, Bernhard Ryffel, Dieudonnée Togbe, Kian Fan Chung

**Affiliations:** ^1^Section of Airways Disease, National Heart and Lung Institute, Imperial College London, London, United Kingdom; ^2^Department of Pulmonary Medicine, Shanghai Chest Hospital, Shanghai Jiao Tong University, Shanghai, China; ^3^Laboratory of Experimental and Molecular Immunology and Neurogenetics (INEM), UMR 7355 CNRS-University of Orleans, Orléans, France; ^4^ArtImmune SAS, Orléans, France

**Keywords:** ozone, oxidative stress, inflammation, empyema, model, COPD

## Abstract

Oxidative stress plays an important role in the pathogenesis of chronic obstructive pulmonary disease (COPD) caused by cigarette smoke and characterized by chronic inflammation, alveolar destruction (emphysema) and bronchiolar obstruction. Ozone is a gaseous constituent of urban air pollution resulting from photochemical interaction of air pollutants such as nitrogen oxide and organic compounds. While acute exposure to ozone induces airway hyperreactivity and neutrophilic inflammation, chronic ozone exposure in mice causes activation of oxidative pathways resulting in cell death and a chronic bronchial inflammation with emphysema, mimicking cigarette smoke-induced COPD. Therefore, the chronic exposure to ozone has become a model for studying COPD. We review recent data on mechanisms of ozone induced lung disease focusing on pathways causing chronic respiratory epithelial cell injury, cell death, alveolar destruction, and tissue remodeling associated with the development of chronic inflammation and AHR. The initial oxidant insult may result from direct effects on the integrity of membranes and organelles of exposed epithelial cells in the airways causing a stress response with the release of mitochondrial reactive oxygen species (ROS), DNA, and proteases. Mitochondrial ROS and mitochondrial DNA activate NLRP3 inflammasome and the DNA sensors cGAS and STING accelerating cell death pathways including caspases with inflammation enhancing alveolar septa destruction, remodeling, and fibrosis. Inhibitors of mitochondrial ROS, NLRP3 inflammasome, DNA sensor, cell death pathways, and IL-1 represent novel therapeutic targets for chronic airways diseases underlined by oxidative stress.

## Introduction

Ozone is a gaseous constituent of urban air pollution that is generated by interaction of rising constituents of air pollution such as nitrogen oxide and organic compounds, induced by sunlight. Early studies have documented the effects of a short acute exposure to ozone in inducing airway inflammation and bronchial hyperreactivity in humans and in various species including mice, rats, guinea pigs, and dogs, at levels of ozone that were much higher than those measured in a highly polluted traffic-dense environment during the summer months.

More than 92% of the world's population are regularly exposed to unhealthy levels of ozone and among the world's 11 most populous countries, population-weighted seasonal ozone concentrations range from about 45 ppb in Brazil to 68 ppb in China. In Europe, the average ozone levels are reported to be highest in Italy with an annual average of 65 ppb (https://www.eea.europa.eu/publications/air-quality-in-europe-2019). Ozone concentration EU targets for the protection of human health has been set not to exceed 60 ppb but these targets are very often exceeded in several EU countries (Table 1). The European Air Quality Report highlights that 17 EU member states and six other reporting countries registered concentrations of ozone above the target value more than 25 times.

**Table 1:** Mean ozone concentration targets and thresholds in Europe.

**Ozone target, threshold, and mean annual values**	**μg/m^**3**^**	**ppm**
EU target (8 h mean)	120	0.060
EU information threshold	180	0.090
EU alert threshold	240	0.120
Level in United Kingdom	80	0.040
Level in France	90	0.045
Level in Germany	100	0.050
Level in Italy	130	0.065

*Adapted from: Air quality in Europe – 2019 report. EU, European Union; ppm, parts per million. (https://www.eea.europa.eu/publications/air-quality-in-europe-2019)*.

Over the recent years, there has been growing evidence from epidemiological and human exposure studies of the detrimental effects of ozone on respiratory health. Thus, short-term changes in ozone levels have been associated with increased mortality (1, 2), and a positive association between ozone and hospital admissions for asthma and COPD in the elderly and between ozone levels and asthma emergency visits in children (3, 4). Indeed, more recent studies have linked long-term exposure to ozone with reduced lung function and an increasing risk of developing emphysema irrespective of being a cigarette smoker (5, 6).

The effects of ozone on inflammation have already been reviewed although these have usually looked at mainly acute exposures (7). One of the long-term interests of the effect of ozone has been to determine whether exposure to ozone can represent a good model for airways disease. Acute exposure to ozone in rats or mice and later in humans have been used as a model of bronchial hyperresponsiveness underlined by a neutrophilic inflammation, the mechanisms of this effect having been reviewed (7). However, it is the chronic exposure to ozone that led to a model of emphysema and chronic airway inflammation that has been of the most interest in recent years (8). This represented the most direct proof that oxidative stress could induce features similar to that seen in the condition labeled as chronic obstructive pulmonary disease (COPD).

In this review, we will consider by what mechanisms this oxidant stress can lead to chronic bronchial inflammation and lung destruction of emphysema, and how models of oxidative stress such as exposure to ozone particularly on a long term basis can inform us on the nature of COPD and can be used to examine potentially new treatments.

## Chronic Obstructive Pulmonary Disease (COPD)

COPD is defined as a common, preventable, and treatable disease that is characterized by persistent respiratory symptoms and airflow obstruction that is due to airway and/or alveolar abnormalities usually caused by significant exposure to noxious particles or gases (9, 10). This is a disease with high degree of morbidity and mortality throughout the world. While initially, this was considered to be a disease mainly found or caused by cigarette smoking, it is now agreed that exposure to biomass fuel and increasingly to environmental pollution can also contribute to the pathogenesis of COPD (11).

Some of the pathological features of smoking-induced COPD consist of severe airflow obstruction which is associated partly to the inflammation in the small airways particularly by neutrophils, macrophages, and lymphocytes (12). In addition, there is airway wall remodeling of the small airways, which involves all the components of the airway wall including epithelium, lamina propria, and airway smooth muscle (13, 14). Finally, there is the presence of emphysema, with the destruction of alveolar airspaces, which has been attributed to an imbalance of protease and anti-protease activity with enhanced protease activity associated with neutrophil activation (15). Airway hyperreactivity (AHR) prevalence and pathology in COPD was found to be present in one out of two COPD patients with its presence influenced by decline in lung function and smoking status (16). As such, AHR can be seen as an independent predictor and a contributing factor to COPD development.

Various pathological pathways have been proposed including increased apoptosis mechanisms (17, 18) in addition to inflammageing and autoimmunity (19). There is growing evidence that COPD may represent a cellular senescence program associated with secretion of pro-inflammatory cytokines that cause chronic inflammation, that leads to the increased levels of reactive oxygen species that can induce oxidative stress (20). Oxidative stress mechanisms can also lead to activation of pro-inflammatory pathways in addition to causing DNA damage (21).

An increasing body of evidence has been accumulating linking COPD with the process of autoimmunity, with the presence of autoantibodies in the sera of COPD patients, and with some of the antibodies correlating with particular disease phenotypes (19). Thus, it has been proposed that autoimmunity may play an important role in the pathogenesis of COPD. Interestingly, ozone-exposed mice for 6 weeks exhibited increased antibody titres to carbonyl-modified protein, as well as activated antigen-presenting cells in lung tissue and splenocytes sensitized to activation of carbonyl-modified protein (22). This is evidence of oxidative stress induced antibodies supporting an auto-immune component, as has been described in COPD with the elevation of carbonyl-modified self-protein that correlated with the severity of disease. Cigarette smoke models have also supported this effect of ozone as has been shown in matrix metalloproteinase 12 (MMP12)-generated elastin fragments serving as a self-antigen and driving the cigarette smoke-induced autoimmune processes in mice that result in a bronchitis-like phenotype and airspace enlargement (23). These elastin autoantibodies and others have been described in advanced COPD (24). The fact that autoantibodies in animal models of COPD such as after ozone exposure are capable of inducing a COPD-like disease phenotype indicate an autoimmune mechanisms perhaps involving B cells, plasma cells, and B cell-rich lymphoid follicles that are present in COPD (25).

## Mechanisms of Ozone-Induced Oxidative Stress on Inflammation and Airway Remodeling

It is quite clear that ozone reacts with cellular membranes and with the epithelial lung lining fluid to generate bioactive mediators that cause oxidative stress, innate immune responses, and signaling (26, 27). When inhaled, there is direct contact between ozone and the first level of cells in the airway surface such as airway and alveolar epithelial cells, and airway macrophages. These cells release reactive oxygen species and various other inflammatory mediators including cytokines and lipids from oxidative damage to the airway epithelium (28, 29). In addition, ozone exposure has been shown to impair macrophage phagocytic and efferocytosis function (28, 30) which can cause prolonged injury and inflammation. The mechanisms underlying these changes have been previously reviewed in terms of the transcriptional effects of ozone in the lungs and airways (31, 32).

### Effects of Ozone Exposure on Oxidative Stress and Intracellular Signaling Pathways

The increased oxidative stress as a direct effect of ozone is often investigated in relation to cellular dysfunction. Ozone affects several intracellular pathways in different cell types, but not always in the same way. Ozone exposure in cultured alveolar epithelial cells results in cytotoxicity but does not always affect the production of cytokines (33). However, when conditioned medium from ozone-exposed alveolar macrophages is added to alveolar epithelial cells, the cytokines chemokine (C-X-C) ligand 1 (CXCL1) and C-C motif chemokine ligand 2 (CCL2) were induced, mediated through interleukin-1α (IL-1α) (34). On the other hand, it has been reported that, although human alveolar macrophages are much more sensitive to ozone than epithelial cells, they do not produce increased amounts of IL-6, IL-8, or fibronectin following ozone exposure. This increased sensitivity of alveolar macrophages was observed in the form of increased cell death with a third of the cells lost after a low ozone exposure dose. However, whether macrophages are more sensitive to ozone *in vivo* is not known, but bronchial airway epithelial cells produce substantially more of all three proteins following ozone exposure, and both IL-6 and fibronectin are secreted vectorially toward the apical side at least in the first 4 and 24 h after ozone exposure (29). Ozone exposure has been shown to induce the expression of intracellular adhesion molecule-1 (ICAM-1) and neutrophil adhesion to human airway epithelial cells, which is mediated through the Canonical Transient Receptor Potential Channel 6 (TRPC6) (35). TRPC6 also impacts neutrophilic inflammation by nuclear factor-κB (NF-κB) activation and therefore promoting transcription of inflammatory mediators (35). Therefore, cellular interactions such as those between alveolar macrophages and, airway and alveolar epithelial cells which are the initial airway cells encountered by ozone may be important in the induction of its effects. The role of TRPC6 during oxidative stress conditions has been investigated in several cell types. Oxidative stress induces TRPC6 expression and function in podocytes (36), HEK293T cells (37), vascular myocytes (38), neutrophils (35), and macrophages (39). In a recent study, TRPC6 in bronchial epithelium cells, was shown to act as an oxidative stress sensor where the TRPC6-mediated calcium cascade leads to the activation of the extracellular signal-regulated kinase (ERK) pathway and inflammation (40). This could be a possible inflammatory response pathway in several cell types in response to ozone, but this has yet to be described in COPD.

Ozone exposure activates several types of innate immune cells which play a role in both the Th1 and Th2 inflammatory responses. While the role of neutrophils as part of the Th1 response is well-established, other innate cell such NK cells (41) and innate lymphoid type 2 (ILC2) cells (42) may also contribute. Surfactant protein D released by exposure to ozone has been shown to stimulate interferon (IFN)-γ by NK cells to initiate IFN-γ, IL-12 feedback circuit (41). Continuous exposure to ozone has been shown to result in the conversion of an initial neutrophilic inflammation to an eosinophilic inflammation in the nasal mucosa of mice (43). This was accompanied by an overexpression of type-2 cytokines in the nasal epithelium. The role of ILC2 cells was confirmed by showing that the epithelial-derived alarmins, IL-33, IL-25, and thymic stromal lymphopoietin (TSLP) that were increased in the nasal epithelium was dependent on ILC-2 cells, but not on adaptive T or B lymphoid cells (44). The involvement of ILC2 cells is also supported by the observation that IL-13, a product of such cells, can augment the effects of single ozone exposure, namely AHR and inflammation (45). In addition, ovalbumin sensitization and challenge can also augment ozone exposure effects (46).

Several intracellular pathways are involved in the effects of ozone exposure. Prolonged exposure to ozone induced gene expression levels of several hypoxia inducible factor-1α (HIF-1α) target genes including histone deacetylase 2 (HDAC2), vascular endothelial growth factor (VEGF), Kelch-like ECH-associated protein 1 (Keap1), and Macrophage migration inhibitory factor (MIF), in addition to a decrease in the antioxidative stress response as indicated by an increase in nuclear erythroid-related factor 2 (Nrf2) activity and protein level (47). The Toll like receptors (TLRs) and downstream adaptor proteins myeloid differentiation marker 88 (MyD88) and toll-interleukin 1 receptor domain containing adaptor protein (TIRAP) appear to be involved in the inflammation response to ozone (48, 49). Ozone is able to induce TLR4 signaling through MyD88 in normal *Tlr4* expressing C3H/HeOuJ mice. This in contrast to C3H/HeJ mice that express a defective dominant negative mutant *Tlr4* gene (50). TLR4 can be activated by ligand binding and subsequently recruits adapter proteins including MyD88. The MyD88-dependant pathway signals through several signal transduction pathways including MAPK, NF-κB, and AP-1 to induce cytokine gene expression contributing to the inflammation response toward ozone. Heath shock protein 70 (HSP70) was identified as a downstream mediator to have a role the TLR4 mediated effects by ozone (50). The ozone-induced oxidative stress response might be involved in the activation of the NACHT, LRR, and PYD domains-containing protein 3 (NLRP3) inflammasome complex (51). Both the acute and chronic exposure of mice to ozone induces lung inflammation through activation of ROS and of the NLRP3 inflammasome with caspase activation (52, 53) and release of the highly inflammatory mediator IL-1β, but also IL-1α (54), which are involved in the ozone injury response (55). Uric acid crystals cause NLRP3-dependent lung inflammation upon injury (56) and therefore it is not excluded that crystals formed during injury may contribute to ozone induced inflammation (57).

How this cytoplasmic multiprotein complex is activated upon ozone exposure remains to be determined. One of the mechanisms might involve reactive oxygen species (ROS) and oxidative stress induced danger signals as reviewed recently (58). The transcriptional effects of ozone and the subsequent impact on airway inflammation have recently been reviewed in an article in this series (31).

### Reactive Oxygen Species-Mediated Barrier Disruption, Inflammation, and Emphysema

Ozone reacts with cellular membranes and with the epithelial lung lining fluid to generate bioactive mediators that cause oxidative stress, innate immune responses, and signaling (26, 27). Ozone-induced ROS at the cell membrane influences membrane integrity with the disruption of tight junction, cell stress, and death with leak of the respiratory barrier within 1–2 h. This first phase is independent of cellular ROS, inflammatory mediators, and inflammatory cells (58). These effects might be the result of ozone exposure induced changes in the expression of claudin (CLDN) proteins which are protein components of tight junctions. In mice exposed to ozone an increased expression levels of CLDN3 and CLDN4 while a reduction in CLDN14 was reported (59). These workers also showed that bronchial epithelial cell integrity was disrupted and that the trans-electrical resistance between cells was decreased leading to cell disintegration. These changes were associated with elevated ROS and increased expression of antioxidant defense system involving Nrf2 and Keap1 (59). Another potential mechanism is lipid peroxidation, which is induced by ozone in human alveolar epithelial cells (60) and in lung surfactant (61). Phospholipids and cholesterol in cell membranes and surfactant can react with ozone to form cytotoxic products which activate second messenger systems involving free arachidonic acid (62), platelet-activating factor (PAF) (63) and prostaglandin E2 (PGE2) (64). Ozone exposure induced changes to cell membrane integrity might involve several pathways, dependent on dose and duration of exposure.

The acute respiratory barrier disruption by ozone has been recently reviewed in an accompanying review in this series (65). This first phase is followed by a robust barrier injury with cell death, protein leak, and influx of ROS expressing myeloid cells including neutrophils, IL-1α and IL-33 production by epithelial and myeloid cells within 6–12 h. Thus, a biphasic response is observed, an immediate direct membrane damage by ROS and a second phase mediated by myeloid cells, which aggravates the damage of the epithelium and inflammation. In support of this hypothesis is the fact that neutrophil depletion attenuates the second phase (58). Oxidative stress peaks at ~18 h after a single ozone exposure during the second phase with increased ROS positive neutrophils and epithelial cells (66). This increase in ROS subsequently causes cell injury, mitochondrial dysfunction, formation, and release of toxic metabolites and even DNA damage. These events have been associated with and contribute to the development of tissue destruction leading to emphysema and lung remodeling.

The toxic effect of ozone was also observed in the distal part of the lung, the alveoli, with death of alveolar epithelial cells with defective repair resulting in enlarged air spaces and emphysema (8). Emphysema upon chronic ozone exposure is commonly found in mice and is dependent on ROS-dependent inflammation (8), but this does not involve IL-17 (67).

Understanding the initial physico-chemical and molecular events of ozone-induced cell membrane injury *in vitro* and *in vivo* is an area of intense research leading to cell death will be important. Inflammation and cell death are also associated with neutrophil extracellular trap (NET) formation with chromatin externalization (68) and release of extracellular DNA, which are inflammatory activating the DNA sensing pathways cGAS/STING as reviewed for lung inflammation (69). Aerosol particles such as silica and likely others including ozone cause extracellular release of nuclear and mitochondrial DNA from dying cells, which drive cGAS/STING-type I interferon dependent inflammation, which is inhibited by DNase administration or blockade of STING (70).

In ozone-exposed mice, ROS-induced cell-death was dependent on interactions of Keap1, the cellular ROS sensor and the phosphatase phosphoglycerate mutase family member 5 (PGAM5) with antioxidant function and apoptosis inducing factor mitochondria associated 1 (AIFM1), a pro-apoptotic factor. At high ROS concentration PGAM5 is released from the complex and activates the pro-apoptotic factor AIFM1 inducing apoptotic pathway resulting in a new form of cell death known as oxeiptosis (71–73). However, the contribution of the different cell death pathways such as necroptosis, apoptosis, and efferocytosis by ozone is yet resolved (74). These cell death pathways are likely to be responsible for the emphysematous process seen on oxidant-induced emphysema.

## Effect of Ozone Exposure on Oxidative Stress and Mitochondrial Dysfunction

Ozone exposure in *ex vivo* and *in vivo* experimental models has focussed recently on the mitochondrial effects of ozone. Mitochondria are double membrane bound organelles that exist in most eukaryotic organisms. Mitochondria play an important role in production of adenosine triphosphate (ATP) and mitochondrial ROS production (75). Dysfunctional mitochondria influence airway contractility, gene expression, oxidative stress, cell proliferation, apoptosis and metabolism, and immune and inflammatory responses that are all implicated in airway diseases including COPD (76).

### Influence of Age on Acute and Chronic Ozone Exposure

The toxic effects of ozone on mitochondria are closely dependent upon the dose and duration of ozone exposure and age of animals (Table 2). Short-term ozone exposure (2 ppm for 8 h or 4 ppm for 4 h) to adult rats caused decreases in mitochondrial O_2_ consumption, mitochondrial ATP synthesis and oxidation of thiol groups in mitochondria in lung, and an increase in lung mitochondrial permeability (77). *In vitro* mitochondrial respiration in lung homogenates and in lung mitochondria from adult rats (2–3 months) was decreased after acute ozone exposure (15 ppm, 20 min). Subacute ozone exposure (0.8 ppm, for 10–20 days) to adult rats led to increased mitochondrial O_2_ consumption in lung homogenates, increased mitochondrial number and mitochondrial respiratory activity in alveolar type II cells in the lung (81). Acute ozone exposure (3 ppm for 8 h) in both adult (4–6 months) and old rats (24–26 months) decreased mitochondrial respiration and mitochondrial O_2_ consumption in isolated lung mitochondria (78). In young (3 weeks) and adult rats (6 months), a more chronic ozone exposure (0.5 ppm, 12 h, 7 days) did not affect lung mitochondrial O_2_ consumption. However, in aged rats (20 months), ozone increased the mitochondrial O_2_ consumption and H_2_O_2_ release, which means increased mitochondrial ROS activity (80). This indicates that ozone may accelerate senescence process in the elderly.

**Table 2:** Dose, duration, and effect of ozone exposures at different ages of rodents.

**Dose**	**Duration**	**Species**	**Age**	**Effect**
**Acute ozone exposure**				
2 ppm	8 h	Rat	Adult	Decrease ATP synthesis and mtO_2_ consumption, increase lung mitochondria permeability (77)
3 ppm	8 h	Rat	Adult	Decreased mitochondrial respiration, decrease mtO_2_ consumption (78)
3 ppm	8 h	Rat	Old	Decreased mitochondrial respiration, decrease mtO_2_ consumption (78)
3 ppm	3 h	Mouse	Adult	Increased mitochondrial ROS, reduced membrane potential, ETC I, III, V proteins, and ATP content (79)
4 ppm	4 h	Rat	Adult	Decrease mtO_2_ consumption (77)
**Chronic ozone exposure**				
0.5 ppm	12 h per day for 7 days	Rat	Young	Increased ROS, decreased ventilatory function (80)
0.5 ppm	12 h per day for 7 days	Rat	Adult	No effects on mitochondria O2 consumption (80)
0.5 ppm	12 h per day for 7 days	Rat	Old	Increased ROS and decreased mitochondria O2 consumption, increased ROS (80)
0.8 ppm	10–20 days	Rat	Adult	Increase number of mitochondria, increase O_2_ consumption rates (81)
2.5 ppm	3 h, twice a week for 6 weeks	Mouse	Adult	Increased mitochondrial ROS, reduced membrane potential, increased ETC protein II, and IV (82)
3 ppm	3 h, twice a week for 6 weeks	Mouse	Adult	Increased mitochondrial ROS, reduced membrane potential, ETC protein and ATP content (79)

*Young = <2 months age, Adult = 2–6 months of age, Old = >6 months of age. ATP, adenosine triphosphate; ETC, electron transport chain; mtO_2_, mitochondrial oxygen consumption; ppm, parts per million; ROS, reactive oxygen species*.

In mice, a single ozone exposure (3 ppm for 3 h) resulted in enhanced cellular and mitochondrial ROS levels and a reduced mitochondrial membrane potential (ΔΨm) in lung. (83). Upon acute and chronic ozone exposures, increased mitochondrial ROS levels, decreased ATP content, decreased electron transport chain (ETC) complex I enzyme activity, and reduced expression of ETC complex I, complex III, and complex V in the lungs of 1- and 6-week ozone-exposed mice (3 ppm, 3 h, twice a week) have been observed. Furthermore, ozone-induced inflammation, airway hyperreactivity, mitochondrial dysfunction, and ROS levels were reduced when mice were administered the mitochondrial antioxidant MitoQ (79). In addition, ozone induced inflammation, increased mitochondrial ROS, and expression of ETC complex II and IV in lung mitochondria in 6-week ozone exposed mice (2.5 ppm, 3 h, twice a week) was reduced by treatment with MitoTEMPO, another mitochondria-targeting antioxidant (82). Therefore, targeting the mitochondrial dysfunction to prevent or treat the ozone-induced associated inflammation, airway hyperresponsiveness, and oxidative stress could be a promising treatment strategy.

AHR is a hallmark of ozone effects on the airways (66, 67) and is exacerbated in allergen-sensitized mice (84–86). AHR is due to a direct effect of ROS on bronchial smooth muscle cells (79), but also neuronal cells (87). The effect of ROS on cell membrane integrity is likely due to a direct disruption of the cell membrane structure which needs further studies (88). This is further supported by the effect of N-acetylcysteine (NAC) in reversing established AHR after chronic exposure to ozone (89). Studies of the airway smooth muscle from mice exposed to ozone showed that the hyperresponsiveness to cholinergic contractile agents can be reproduced *in vitro* and that this oxidant stress-induced hyperresponsiveness was dependent on the activation of the p38 mitogen-activated kinase (MAPK) and inhibited by corticosteroids (52). The AHR induced by chronic ozone exposure was also dependent on IL-17 (67), as was also shown by Pichavant et al. who reported that NK cells producing IL-17 was important for the maintenance of ozone-induced AHR (86). The pulmonary inflammation induced by subacute ozone exposure has been reported to require γδ T cells and tumor necrosis factor-α (TNFα)-dependent recruitment of IL-17A + γδ T cells to the lung (90).

## Therapeutic Strategies Targeting Inflammation, Oxidative Stress, and Mitochondria

Several studies have investigated the effects of novel treatment strategies relating to the effects of ozone exposure in animal models (Table 3). Targeting inflammation, oxidative stress, and mitochondria could be part of future treatment strategies for COPD.

**Table 3:** Effect of different treatment strategies in ozone-exposed animal models.

**Compound**	**Target**	**Effect**	**References**
Apocynin	Cellular ROS	Inhibition epithelial cell proliferation	(91)
Corticosteroids	MAPK	Inhibition of inflammation, AHR	(67)
H_2_S	Cellular and mitochondrial ROS	Inhibition of inflammation, ROS, and emphysema	(92, 93)
iPSC-MSC	Lung	Inhibition of cellular and mitochondrial ROS, normal membrane potential	(83)
ISO-1	MIF cytokine	Inhibition of inflammation and AHR	(94)
MitoTEMPO	Mitochondria	Inhibition mitochondrial ROS, normal complex protein expression	(82)
MitoQ	Mitochondria	Inhibition of inflammation, AHR, mitochondrial ROS and mitochondrial dysfunction	(79)
VX-765	NLRP3 inflammasome	Inhibition of inflammation and AHR Inhibition of inflammation, AHR, emphysema	(53, 82)

*AHR, airway hyperreactivity; H_2_S, hydrogen sulfide; iPSC-MSC, induced pluripotent stem cell-derived mesenchymal stem cell; ISO-1, (S,R)3-(4-hydroxyphenyl)-4,5-dihydro-5-isoxazole acetic acid methyl ester; MAPK, mitogen activated kinase; MIF, macrophage migration inhibitory factor; NLRP3, NACHT, LRR, and PYD domains-containing protein 3; ROS, reactive oxygen species; VX-765, Belnacasan, caspase-1 inhibitor*.

### Corticosteroids and ISO-1

Corticosteroids can attenuate the single exposure effects of ozone including AHR and lung inflammation (95–97). This includes the inhibition of expression of macrophage inflammatory protein 2 (MIP-2), inducible nitric oxidase synthase (iNOS) (98, 99) and NFκB (96), and the increased proliferation of the airway epithelium (91). However, chronic exposure to ozone itself can induce a state of corticosteroid insensitivity similar to what has been observed in COPD with reduced or little effect in preventing AHR, inflammation, and emphysema (94, 100). Corticosteroid insensitivity occurs in COPD through mechanisms induced by reactive oxygen species (101, 102). Several such mechanisms have been postulated, including a reduction in HDAC2 activity and expression, impaired corticosteroid activation of the glucocorticoid receptor (GR) and increased pro-inflammatory signaling pathways. Therefore, the chronic model of ozone exposure represents a good model of COPD to study the mechanisms of corticosteroid insensitivity.

Macrophage migration inhibitory factor (MIF) has been implicated as a driver of inflammation in COPD, and possibly as a driver of corticoidsteroid insensitivity in COPD. Using (S,R)3-(4-hydroxyphenyl)-4,5-dihydro-5-isoxazole acetic acid methyl ester (ISO-1) which inhibits MIF tautomerase activity, the corticosteroid-insensitive lung inflammation and AHR after chronic ozone exposure was blocked (94). Thus, inhibition of MIF which is elevated in COPD may provide a novel anti-inflammatory approach in COPD. However, the contribution of mitogen-activated protein kinase phosphatase-1 (MKP-1) that has been proposed to underlie CS insensitivity in COPD was found to be negligible in the chronic ozone model (100). Single ozone exposure aggravated airway inflammation, airway remodeling, activation of p38 MAPK, and downregulation of MKP-1 in ovalbumin (OVA)-sensitized and -challenged mice, an effect that was ineffectively controlled by corticosteroids (46), this also supported the role of p38 MAPK activation as a likely pathway involved in corticosteroid insensitivity (103).

### Hydrogen Sulfide (H_2_S)

Hydrogen sulfide (H_2_S), a metabolic product of methionine, is synthesized from L-cysteine primarily by three key enzymes: cystathionine-c-lyase (CGL), cystathionine-b-synthetase (CBS) and 3-mercaptypyruvate sulfurtransferase (MPST). Identified as the third gasotransmitter, along with nitric oxide and carbon monoxide, H_2_S modulates a variety of physiological functions including anti-oxidative stress, anti-senescence/aging, and anti-apoptotic effects (104–106). H_2_S has been proposed as serving as a potent antioxidant through reactive oxygen species/reactive nitrogen species scavenging, or through post-translational modification of proteins by addition of a thiol (-SH) group onto reactive cysteine residues (107). In the lungs, H_2_S suppresses the airway smooth muscle proliferation and cytokine release, an effect that is less effective in muscle from COPD patients (108).

In addition, H_2_S content is reduced in the lungs of smokers and COPD patients (109), and H_2_S attenuates nicotine-induced endoplasmic reticulum stress and apoptosis in bronchial epithelial 16HBE cells (110). H_2_S is able to prevent and treat the development of inflammation, AHR, and oxidative stress in acute ozone-exposed mice (92). In addition, the ozone-induced increase in p38 MAK signaling was reduced in mice treated with H_2_S indicating that this intracellular signaling pathway might be involved (92). In chronic ozone-exposed mice, H_2_S is also able to prevent the inflammation, AHR, and remodeling of the lung but is not able to reverse these hallmarks of the model (93). Induction of p38 MAPK signaling is also reversed in both treatment strategies in the chronic ozone exposure model. In addition to this, the activation of the NLRP3 inflammasome and the ratio between cleaved caspase-1 to pro-caspase-1 were positively correlated with changes in lung function parameters and structural changes in the lung. H_2_S was able to prevent and treat the changes observed in NLRP3 activation (93). Taken together, the main difference between the acute and chronic ozone exposure models on the treatment effects of H_2_S on the ozone-induced changes suggests that H_2_S treatment only affects the damage inducing pathways (oxidative stress) and not the regenerative pathways of the lung (92, 93). It is of interest that H_2_S donor NaHS significantly inhibits cigarette smoke-induced mitochondrial dysfunction, oxidative damage, cell senescence, and apoptosis in alveolar epithelial A549 cells (111). These findings provide novel mechanisms underlying the protection of H_2_S against ozone and cigarette smoke-induced COPD and suggest that H_2_S donors targeted toward mitochondria may be beneficial in the treatment of COPD.

### NLRP3 Inflammasome Inhibitor, Belnacasan (VX-765)

In addition to H_2_S, Belnacasan or VX-765 inhibits NLRP3 inflammasome activation effects by inhibiting caspase-1. In acute ozone exposed mice VX-765 is able to prevent bronchoalveolar lavage (BAL) inflammatory markers, and AHR. Mitochondrial oxidative stress was reduced and this was associated with lower expression levels of dynamin-related protein 1 (DRP1) and mitochondrial fission factor (MFF), and increased expression of Mitofusin 2 (MFN2) proteins involved in mitochondrial fission and fusion, respectively (53). Similar effects were observed in chronic ozone exposed mice were VX-765 is able to prevent inflammation, emphysema, airway remodeling, and oxidative stress while it decreased the expression of the fission protein DRP1 and MFF with affecting proteins involved in fusion dynamics. (82). Mitochondrial oxidative stress and NLRP3 inflammasome were driving ozone-induced inflammation processes and targeting these specifically might have therapeutic value in COPD.

### Mitochondrial-Targeted Antioxidants

Apocynin, reduced nicotinamide adenine dinucleotide phosphate (NADPH) oxidase inhibitor decreased the proliferation in bronchial epithelium after an acute exposure to ozone but not the inflammation (91). Ozone-induced inflammation, airway hyperreactivity, mitochondrial dysfunction, and ROS levels were reduced when chronically-exposed mice were pre-administered the mitochondrial directed antioxidant, MitoQ (79). Similarly, ozone-induced inflammation, increased mitochondrial ROS, and expression of ETC complex II and IV in lung mitochondria in 6-week ozone exposed mice was reduced by treatment with mitoTEMPO, another mitochondria-targeting antioxidant (82). However, airway remodeling and airflow obstruction were not (82). Similarly, in single exposure to ozone, mitoTEMPO inhibited mitochondrial ROS without affecting inflammation and bronchial hyperresponsiveness (53).

Mucolytic/antioxidant agents such as erdosteine, carbocysteine, and NAC reduced the risk of acute exacerbations in patients with COPD (112). In the chronic ozone exposure model, preventive NAC reduced the number of BAL macrophages and airway smooth muscle (ASM) mass while therapeutic NAC reversed AHR, and reduced ASM mass and apoptotic cells (89). Thus, NAC could represent a treatment for protecting against the oxidative effects of ozone and other pollutants, as well as an agent for reducing exacerbations of COPD.

### Downstream Signaling Pathways

As documented above, several intracellular pathways have been implicated in the effects of single or multiple exposures to ozone in the mouse. These pathways are related to the control of several key transcription regulatory factors including NF-κB, antioxidant factors such as Nrf2, the p38 MAPK, and priming of the immune system by up-regulating toll-like receptor expression. Thus, in the single ozone exposure model, AHR and inflammation was inhibited by a c-jun NH2 terminal kinase (JNK) inhibitor (SP600125) (113), p38 MAPK inhibitor (SD282) (46, 114) and NF-κB inhibitor (115). VX-765, an inhibitor of NLRP3 inflammasome, prevented lung inflammation and AHR caused by acute exposure to ozone (53), and also inhibited lung inflammation and emphysema from chronic exposure (82).

### iPSC-MSC Mitochondrial Transfer

Mitochondrial transfer from induced pluripotent stem cell-derived mesenchymal stem cell (iPSC-MSC) offered protection against oxidative stress-induced mitochondrial dysfunction in human airway smooth muscle cells (ASMC) and in mouse lungs exposed to ozone while reducing airway inflammation and hyperresponsiveness (83). Direct co-culture of ASMCs with iPSC-MSCs protected the former from cigarette smoke-induced mitochondrial ROS production, mitochondrial depolarization, and apoptosis. When ASMCs were exposed to supernatants from iPSC-MSCs or transwell inserts with iPSC-MSCs, only cigarette smoke-induced mitochondrial ROS, but not mitochondrial depolarization and apoptosis in ASMCs, were improved, indicating that soluble factors from iPSC-MSCs reduced production of mitochondrial ROS. When there was direct contact between iPSC-MSCs and ASMCs, mitochondria were transferred from iPSC-MSCs to ASMCs, possibly through formation of tunneling nanotubes, an effect that was enhanced by cigarette smoke medium (CSM) treatment. iPSC-MSCs prevented, but did not reverse, ozone-induced mitochondrial dysfunction, AHR, and airway inflammation in the mouse model of single ozone exposure, an effect resulting from direct interaction and mitochondrial transfer between iPSC-MSCs and airway cells. Therefore, transfer of mitochondria from IPSC-MSC cells to replace damaged mitochondria by oxidative stress may present a novel approach to treating conditions such as COPD.

## Conclusion

**Figure 1:**
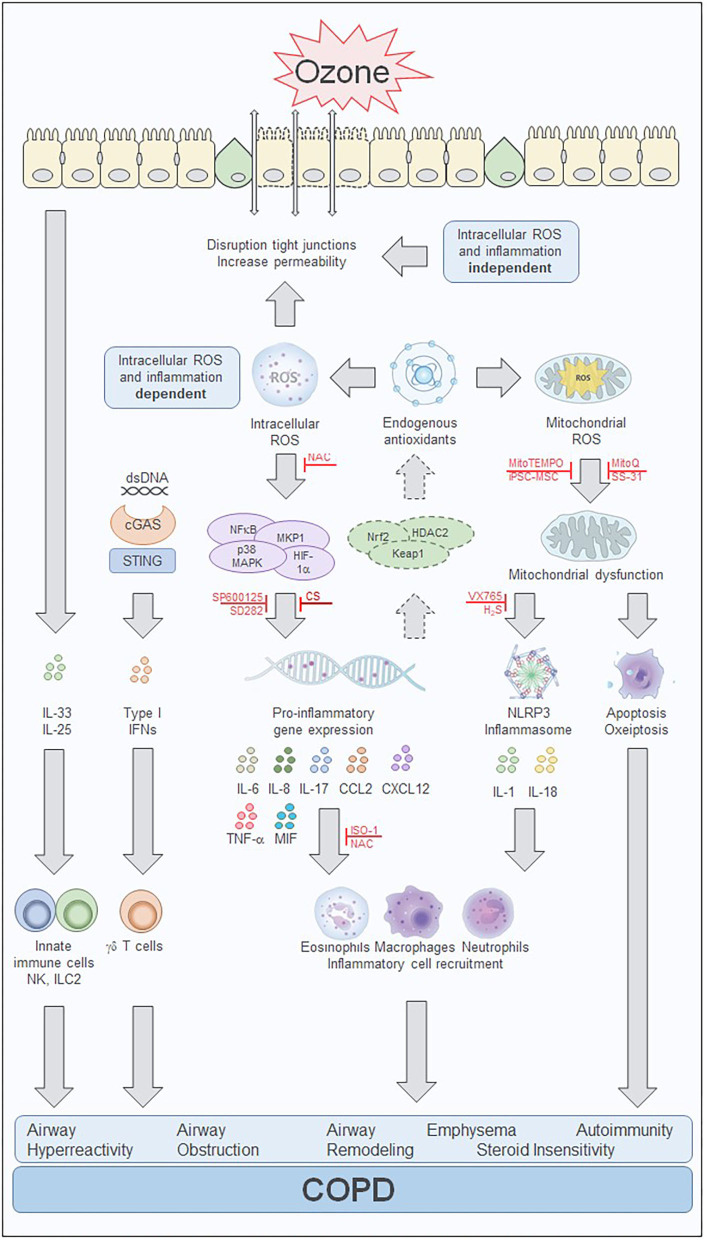
Ozone-mediated effects on intracellular pathways involved in cell injury and inflammation. Overview of the effects of ozone in *in vitro* and *in vivo* models. The biphasic response to ozone starts with an immediate intracellular reactive oxygen species (ROS) and inflammation independent phase that is induced by extracellular oxidative stress. ROS induces membrane damage with changes in cell membrane integrity, disruption of tight junctions, epithelial cell stress, and death. Inflammatory mediators including IL-25 and IL-33 are released and attract innate immune cells such as natural killer (NK) cells and innate lymphoid type 2 (ILC2) cells. Cell death and release of dsDNA can induce an interferon (IFN) inflammation response via the cGAS/STING pathway. Processes activated during the intracellular ROS and inflammation-dependent phase of the effects of ozone include transcription factor-mediated inflammatory response and the activation of the antioxidant defense mechanism. Several inhibitors have been shown to prevent or treat the pro-inflammatory gene expression and subsequently inhibit the inflammatory response, including the JNK inhibitor, SP600125, and the p38 MAPK inhibitor, SD282. In addition, activation of the endogenous antioxidant defense system involving HADC2, Keap1, and Nrf2 may be sufficient to counteract the oxidant stress during acute ozone exposure but may be overwhelmed during chronic ozone exposure. Treatment with the antioxidant N-acetylcysteine (NAC) also reduces the inflammatory response, by scavenging of intracellular ROS with subsequent reduction of cytokine and chemokine production. Mitochondrial oxidative stress and mitochondrial dysfunction contribute to apoptotic processes and the activation of the NLRP3 inflammasome further enhancing the inflammatory response. Several treatment strategies targeting the mitochondria have been able to reduce or prevent the mitochondrial oxidative-induced dysfunction. These include several mitochondrial-targeted antioxidants such as MitoQ, MitoTEMPO, and SS-31. In addition, stem cell therapy with induced pluripotent stem cell-derived mesenchymal stem cells (iPSC-MSCs) prevented ozone-induced mitochondrial dysfunction and inflammation which may result from direct interaction and mitochondrial transfer between the iPSC-MSCs and airway cells. Treatment with the caspase-1 inhibitor VX765 and hydrogen sulfide (H_2_S) prevent the activation of the inflammasome and reduces inflammation and mitochondrial oxidative stress. The MIF inhibitor ISO-1 blocks the ozone exposure induced inflammation and airway hyperreactivity and might have an impact on the corticosteroid insensitivity present in chronic ozone exposed lungs. Corticosteroids reduce inflammation induced by acute ozone exposures but fail to affect these processes in the steroid insensitive chronic ozone exposed lung. The ozone exposure driven intracellular processes contribute to the inflammatory cytokine and chemokine production, immune cell recruitment, and eventually the development of airway hyperreactivity, airway obstruction, airway remodeling, emphysema, autoimmunity, and steroid insensitivity which are hallmarks of COPD. cGAS, cyclic GMP-AMP synthase; COPD, chronic obstructive pulmonary disease; dsDNA, double stranded DNA; H2S, hydrogen sulfide; HDAC2, histone deacetylase 2; HIF-1α, hypoxia inducible factor subunit 1α; IFN, interferon; IL, interleukin; ILC2, innate lymphoid cells type 2; iPSC-MSC, induced pluripotent stem cell-derived mesenchymal stem cell; ISO-1, (S,R)3-(4-hydroxyphenyl)-4,5-dihydro-5-isoxazole acetic acid methyl ester; Keap1, Kelch-like ECH associated protein 1; MAPK, mitogen activated kinase; MKP-1, mitogen-activated protein kinase phosphatase-1; MIF, macrophage migration inhibitory factor; MKP-1, mitogen-activated protein kinase phosphatase-1; NAC, N-acetylcysteine; NFκB, nuclear factor κB; NK, natural killer cell; NLRP3, NACHT, LRR, and PYD domains-containing protein 3; Nrf2, nuclear factor (erythroid-derived 2)-like 2; ROS, reactive oxygen species; SD282, p38 MAPK inhibitor; SP600125, c-jun NH2 terminal kinase (JNK) inhibitor; SS-31, d-Arg-2', 6'-dimethyltyrosine-Lys-Phe-NH2 mitochondrial antioxidant; STING, stimulator of interferon genes; TNF-α, tumor necrosis factor -α; VX765, Belnacasan, caspase 1 inhibitor.

As an important component of air pollution, ozone has been closely related to the development of COPD. The link comes from two sides: on the one hand, chronic exposure to ozone in murine model of lung inflammation and emphysema and on the other, long-term exposure to ambient air pollutant such as ozone, has been associated with increases in emphysema evaluated by computed tomographic imaging with chronic airflow obstruction. The mechanisms of ozone-induced lung and airway changes are the release of inflammatory factors such as IL-1α, IL-6, IL-8, CXCL-12, CCL2, ICAM-1, KEAP-1, and MIF, the activation of intracellular pathways such as the MAPK pathway, TLR, cell death pathways, NLRP3 inflammasome, and NF-κB, the induction of oxidative stress through a decrease in the antioxidative response and an increase in the production of ROS, with a detrimental effect on mitochondrial function such as increased mitochondrial ROS, decreased ATP content and abnormal ETC complex (Figure 1). Other mechanisms include the disruption of airway epithelial barrier, the development of AHR and emphysema and the state of CS insensitivity. Inhibitors of mitochondrial ROS, NLRP3 inflammasome, DNA sensor, cell death pathways and IL-1, including hydrogen sulfide, apocynin, mitochondrial-targeted antioxidants such as MitoQ and MitoTEMPO, mucolytic/antioxidant agents such as NAC, VX765, and iPS-MSCs mitochondrial transfer represent novel therapeutic options for treating oxidative stress-induced COPD.

## Author Contributions

CW, FL, DT, and KC wrote the review. KC and BR inspired the work and corrected the manuscript. All authors have read the contribution.

## Conflict of Interest

DT was employed by the company ArtImmune SAS. The remaining authors declare that the research was conducted in the absence of any commercial or financial relationships that could be construed as a potential conflict of interest.

## Abbreviations

16HBE: human bronchial epithelial cell line
AHR: airways hyperreactivity
AIFM1: mitochondrial apoptosis inducing factor 1
AP-1: activator protein 1
ASMC: airway smooth muscle cell
ATP: adenosine triphosphate
BAL: bronchoalveolar lavage
cGAS: cyclic GMP-AMP synthase
CBS: cystathionine-beta-synthetase
CCL2: C-C motif chemokine ligand 2
CGL: cystathionine-gamma-lyase
CLDN: claudin
COPD: chronic obstructive pulmonary disease
CS: corticosteroid
CSM: cigarette smoke medium
CXCL1: chemokine (C-X-C) ligand 1
ΔΨm: mitochondrial membrane potential
DRP1: dynamin-related protein 1
ERK: extracellular signal-regulated kinase
ETC: electron transport chain
GR: glucocorticoid receptor
H_2_O_2_: hydrogen peroxide
H_2_S: hydrogen sulfide
HDAC2: histone deacetylase 2
HIF-1α: hypoxia inducible factor subunit 1α
ICAM1: intracellular adhesion molecule 1
IFNγ: interferon gamma
IL: interleukin
ILC2: innate lymphoid cells type 2
iNOS: inducible nitric oxide synthase
iPSC-MSC: induced pluripotent stem cell-derived mesenchymal stem cell
ISO-1: (S,R)3-(4-hydroxyphenyl)-4,5-dihydro-5-isoxazole acetic acid methyl ester
JNK: c-jun NH2 terminal kinase
KEAP1: Kelch-like ECH associated protein 1
MAPK: mitogen activated kinase
MFF: mitochondrial fission factor
MFN2: mitofusin 2
MIF: macrophage migration inhibitory factor
MIP-2: macrophage inflammatory protein 2
MKP-1: mitogen-activated protein kinase phosphatase-1
MMP-12: matrix metalloproteinase 12
MPST: 3-mercaptypyruvate sulfurtransferase
MyD88: myeloid differentiation marker 88
NAC: N-acetylcysteine
NADPH: nicotinamide adenine dinucleotide phosphate
NaHS: sodium hydrosulfide
NET: neutrophil extracellular trap
NFκB: nuclear factor κB
NK: natural killer cell
NLRP3: NACHT, LRR and PYD domains-containing protein 3
Nrf2: nuclear factor (erythroid-derived 2)-like 2
OVA: ovalbumin
PAF: platelet-activating factor
PGAM5: phosphoglycerate mutase family, member 5
PGE2: prostaglandin E2
ppm: parts per million
ROS: reactive oxygen species
SS-31: d-Arg-2′,6′-dimethyltyrosine-Lys-Phe-NH_2_ mitochondrial antioxidant
STING: stimulator of interferon genes
TIRAP: Toll-Interleukin 1 receptor (TIR) domain containing adaptor protein
TLR: toll-like receptor
TNF-α: tumor necrosis factor-α
TRPC6: canonical transient receptor potential channel 6
TSLP: thymic stromal lymphopoietin
VEGF: vascular endothelial growth factor.

## References

[1] Bell ML McDermott A Zeger SL Samet JM Dominici F. Ozone and short-term mortality in 95 US urban communities, 1987-2000. JAMA. (2004) 292:2372–8. 10.1001/jama.292.19.2372 15547165 PMC3546819

[2] Vicedo-Cabrera AM Sera F Liu C Armstrong B Milojevic A Guo Y. Short term association between ozone and mortality: global two stage time series study in 406 locations in 20 countries. BMJ. (2020) 368:m108. 10.1136/bmj.m108 32041707 PMC7190035

[3] Gao H Wang K Au WW Zhao W Xia ZL. A systematic review and meta-analysis of short-term ambient ozone exposure and COPD hospitalizations. Int J Environ Res Public Health. (2020) 17:2130. 10.3390/ijerph17062130 32210080 PMC7143242

[4] Halonen JI Lanki T Yli-Tuomi T Kulmala M Tiittanen P Pekkanen J. Urban air pollution, and asthma and COPD hospital emergency room visits. Thorax. (2008) 63:635–41. 10.1136/thx.2007.091371 18267984

[5] Paulin LM Gassett AJ Alexis NE Kirwa K Kanner RE Peters S. Association of long-term ambient ozone exposure with respiratory morbidity in smokers. JAMA Intern Med. (2020) 180:106–15. 10.1001/jamainternmed.2019.5498 31816012 PMC6902160

[6] Wang M Aaron CP Madrigano J Hoffman EA Angelini E Yang J. Association between long-term exposure to ambient air pollution and change in quantitatively assessed emphysema and lung function. JAMA. (2019) 322:546–56. 10.1001/jama.2019.10255 31408135 PMC6692674

[7] Bromberg PA. Mechanisms of the acute effects of inhaled ozone in humans. Biochim Biophys Acta. (2016) 1860:2771–81. 10.1016/j.bbagen.2016.07.015 27451958

[8] Triantaphyllopoulos K Hussain F Pinart M Zhang M Li F Adcock I. A model of chronic inflammation and pulmonary emphysema after multiple ozone exposures in mice. Am J Physiol Lung Cell Mol Physiol. (2011) 300:L691–700. 10.1152/ajplung.00252.2010 21355040 PMC3094024

[9] Agusti A Hogg JC Singh D Agusti A Anzueto A Barnes PJ. Update on the pathogenesis of chronic obstructive pulmonary disease global strategy for the diagnosis, management, and prevention of chronic obstructive lung disease: the GOLD science committee report 2019. N Engl J Med. (2019) 381:1248–56. 10.1056/NEJMra1900475 31553836

[10] Chung KF Adcock IM. Multifaceted mechanisms in COPD: inflammation, immunity, and tissue repair and destruction. Eur Respir J. (2008) 31:1334–56. 10.1183/09031936.00018908 18515558

[11] Salvi SS Barnes PJ. Chronic obstructive pulmonary disease in non-smokers. Lancet. (2009) 374:733–43. 10.1016/S0140-6736(09)61303-9 19716966

[12] Hogg JC Chu F Utokaparch S Woods R Elliott WM Buzatu L. The nature of small-airway obstruction in chronic obstructive pulmonary disease. N Engl J Med. (2004) 350:2645–53. 10.1056/NEJMoa032158 15215480

[13] Koo HK Vasilescu DM Booth S Hsieh A Katsamenis OL Fishbane N. Small airways disease in mild and moderate chronic obstructive pulmonary disease: a cross-sectional study chronic cough as a novel phenotype of chronic obstructive pulmonary disease. Lancet Respir Med. (2018) 6:591–602. 10.1016/S2213-2600(18)30196-6 30072106

[14] McDonough JE Yuan R Suzuki M Seyednejad N Elliott WM Sanchez PG. Small-airway obstruction and emphysema in chronic obstructive pulmonary disease. N Engl J Med. (2011) 365:1567–75. 10.1056/NEJMoa1106955 22029978 PMC3238466

[15] Barnes PJ. Chronic obstructive pulmonary disease. N Engl J Med. (2000) 343:269–80. 10.1056/NEJM200007273430407 10911010

[16] Scichilone N Battaglia S La Sala A Bellia V. Clinical implications of airway hyperresponsiveness in COPD. Int J Chron Obstruct Pulmon Dis. (2006) 1:49–60. 10.2147/copd.2006.1.1.49 18046902 PMC2706603

[17] Tuder RM Zhen L Cho CY Taraseviciene-Stewart L Kasahara Y Salvemini D. Oxidative stress and apoptosis interact and cause emphysema due to vascular endothelial growth factor receptor blockade. Am J Respir Cell Mol Biol. (2003) 29:88–97. 10.1165/rcmb.2002-0228OC 12600822

[18] Tan WSD Shen HM Wong WSF. Dysregulated autophagy in COPD: a pathogenic process to be deciphered. Pharmacol Res. (2019) 144:1–7. 10.1016/j.phrs.2019.04.005 30953685

[19] Caramori G Ruggeri P Di Stefano A Mumby S Girbino G Adcock IM. Autoimmunity and COPD: clinical implications. Chest. (2018) 153:1424–31. 10.1016/j.chest.2017.10.033 29126842

[20] Zuo L Prather ER Stetskiv M Garrison DE Meade JR Peace TI. Inflammaging and oxidative stress in human diseases: from molecular mechanisms to novel treatments. Int J Mol Sci. (2019) 20:4472. 10.3390/ijms20184472 31510091 PMC6769561

[21] Chung KF Marwick JA. Molecular mechanisms of oxidative stress in airways and lungs with reference to asthma and chronic obstructive pulmonary disease. Ann N Y Acad Sci. (2010) 1203:85–91. 10.1111/j.1749-6632.2010.05600.x 20716288

[22] Kirkham PA Caramori G Casolari P Papi AA Edwards M Shamji B. Oxidative stress-induced antibodies to carbonyl-modified protein correlate with severity of chronic obstructive pulmonary disease. Am J Respir Crit Care Med. (2011) 184:796–802. 10.1164/rccm.201010-1605OC 21965015 PMC3398415

[23] Zhou JS Li ZY Xu XC Zhao Y Wang Y Chen HP. Cigarette smoke-initiated autoimmunity facilitates sensitisation to elastin-induced COPD-like pathologies in mice. Eur Respir J. (2020). 10.1183/13993003.00404-2020. [Epub ahead of print]. 32366484

[24] Byrne R Todd I Tighe PJ Fairclough LC Caramori G Ruggeri P. Autoantibodies in chronic obstructive pulmonary disease: a systematic review autoimmunity and COPD: clinical implications. Immunol Lett. (2019) 214:8–15. 10.1016/j.imlet.2019.08.007 31472176

[25] Wen L Krauss-Etschmann S Petersen F Yu X. Autoantibodies in chronic obstructive pulmonary disease. Front Immunol. (2018) 9:66. 10.3389/fimmu.2018.00066 29422903 PMC5788885

[26] Mudway IS Kelly FJ. Ozone and the lung: a sensitive issue. Mol Aspects Med. (2000) 21:1–48. 10.1016/S0098-2997(00)00003-0 10804262

[27] Pryor WA. Mechanisms of radical formation from reactions of ozone with target molecules in the lung. Free Radic Biol Med. (1994) 17:451–65. 10.1016/0891-5849(94)90172-4 7835752

[28] Becker S Madden MC Newman SL Devlin RB Koren HS Devlin RB. Modulation of human alveolar macrophage properties by ozone exposure *in vitro*. Toxicol Appl Pharmacol. (1991) 110:403–15. 10.1016/0041-008X(91)90042-D 1658983

[29] Devlin RB McKinnon KP Noah T Becker S Koren HS. Ozone-induced release of cytokines and fibronectin by alveolar macrophages and airway epithelial cells. Am J Physiol. (1994) 266:L612–9. 10.1152/ajplung.1994.266.6.L612 8023949

[30] Devlin RB Folinsbee LJ Biscardi F Hatch G Becker S Maden MC Inflammation and cell damage induced by repeated exposure of humans to ozone. Inhal Toxicol. (1997) 9:211–2325. 10.1080/089583797198222

[31] Mumby S Chung KF Adcock IM. Transcriptional effects of ozone and impact on airway inflammation. Front Immunol. (2019) 10:1610. 10.3389/fimmu.2019.01610 31354743 PMC6635463

[32] Tovar A Smith GJ Thomas JM Crouse WL Harkema JR Kelada SNP. Transcriptional profiling of the murine airway response to acute ozone exposure. Toxicol Sci. (2020) 173:114–30. 10.1093/toxsci/kfz219 31626304 PMC6944221

[33] Manzer R Wang J Nishina K McConville G Mason RJ. Alveolar epithelial cells secrete chemokines in response to IL-1beta and lipopolysaccharide but not to ozone. Am J Respir Cell Mol Biol. (2006) 34:158–66. 10.1165/rcmb.2005-0205OC 16239643 PMC2644180

[34] Manzer R Dinarello CA McConville G Mason RJ. Ozone exposure of macrophages induces an alveolar epithelial chemokine response through IL-1alpha. Am J Respir Cell Mol Biol. (2008) 38:318–23. 10.1165/rcmb.2007-0250OC 17901407 PMC2258451

[35] Chen QZ Zhou YB Zhou LF Fu ZD Wu YS Chen Y. TRPC6 modulates adhesion of neutrophils to airway epithelial cells via NF-kappaB activation and ICAM-1 expression with ozone exposure. Exp Cell Res. (2019) 377:56–66. 10.1016/j.yexcr.2019.02.013 30779919

[36] Anderson M Roshanravan H Khine J Dryer SE. Angiotensin II activation of TRPC6 channels in rat podocytes requires generation of reactive oxygen species. J Cell Physiol. (2014) 229:434–42. 10.1002/jcp.24461 24037962

[37] Gibon J Tu P Bohic S Richaud P Arnaud J Zhu M. The over-expression of TRPC6 channels in HEK-293 cells favours the intracellular accumulation of zinc. Biochim Biophys Acta. (2011) 1808:2807–18. 10.1016/j.bbamem.2011.08.013 21864503

[38] Ding Y Winters A Ding M Graham S Akopova I Muallem S. Reactive oxygen species-mediated TRPC6 protein activation in vascular myocytes, a mechanism for vasoconstrictor-regulated vascular tone. J Biol Chem. (2011) 286:31799–809. 10.1074/jbc.M111.248344 21768109 PMC3173128

[39] Finney-Hayward TK Popa MO Bahra P Li S Poll CT Gosling M. Expression of transient receptor potential C6 channels in human lung macrophages. Am J Respir Cell Mol Biol. (2010) 43:296–304. 10.1165/rcmb.2008-0373OC 19843708

[40] Chen Q Zhou Y Zhou L Fu Z Yang C Zhao L TRPC6-dependent Ca(2+) signaling mediates airway inflammation in response to oxidative stress via ERK pathway. Cell Death Dis. (2020) 11:170 10.1038/s41419-020-2678-7 32139669 PMC7058000

[41] Ge MQ Kokalari B Flayer CH Killingbeck SS Redai IG MacFarlane AW Cutting edge: role of NK cells and surfactant protein D in dendritic cell lymph node homing: effects of ozone exposure. J Immunol. (2016) 196:553–7. 10.4049/jimmunol.1403042 26673133 PMC4707083

[42] Harkema JR Wagner JG. Innate lymphoid cell-dependent airway epithelial and inflammatory responses to inhaled ozone: a new paradigm in pathogenesis. Toxicol Pathol. (2019) 47:993–1003. 10.1177/0192623319873872 31537180 PMC6910988

[43] Ong CB Kumagai K Brooks PT Brandenberger C Lewandowski RP Jackson-Humbles DN. Ozone-induced type 2 immunity in nasal airways. Development and lymphoid cell dependence in mice. Am J Respir Cell Mol Biol. (2016) 54:331–40. 10.1165/rcmb.2015-0165OC 26203683

[44] Kumagai K Lewandowski RP Jackson-Humbles DN Buglak N Li N White K. Innate lymphoid cells mediate pulmonary eosinophilic inflammation, airway mucous cell metaplasia, and type 2 immunity in mice exposed to ozone. Toxicol Pathol. (2017) 45:692–704. 10.1177/0192623317728135 28891433

[45] Williams AS Nath P Leung SY Khorasani N McKenzie AN Adcock IM. Modulation of ozone-induced airway hyperresponsiveness and inflammation by interleukin-13. Eur Respir J. (2008) 32:571–8. 10.1183/09031936.00121607 18417511

[46] Liang L Li F Bao A Zhang M Chung KF Zhou X. Activation of p38 mitogen-activated protein kinase in ovalbumin and ozone-induced mouse model of asthma. Respirology. (2013) 18 (Suppl. 3):20–9. 10.1111/resp.12189 24188200

[47] Wiegman CH Li F Clarke CJ Jazrawi E Kirkham P Barnes PJ. A comprehensive analysis of oxidative stress in the ozone-induced lung inflammation mouse model. Clin Sci. (2014) 126:425–40. 10.1042/CS20130039 24040961

[48] Li Z Potts-Kant EN Garantziotis S Foster WM Hollingsworth JW. Hyaluronan signaling during ozone-induced lung injury requires TLR4, MyD88, and TIRAP. PLoS ONE. (2011) 6:e27137. 10.1371/journal.pone.0027137 22073274 PMC3208559

[49] Williams AS Leung SY Nath P Khorasani NM Bhavsar P Issa R. Role of TLR2, TLR4, and MyD88 in murine ozone-induced airway hyperresponsiveness and neutrophilia. J Appl Physiol. (2007) 103:1189–95. 10.1152/japplphysiol.00172.2007 17626835

[50] Bauer AK Rondini EA Hummel KA Degraff LM Walker C Jedlicka AE. Identification of candidate genes downstream of TLR4 signaling after ozone exposure in mice: a role for heat-shock protein 70. Environ Health Perspect. (2011) 119:1091–7. 10.1289/ehp.1003326 21543283 PMC3237361

[51] Michaudel C Couturier-Maillard A Chenuet P Maillet I Mura C Couillin I. Inflammasome, IL-1 and inflammation in ozone-induced lung injury. Am J Clin Exp Immunol. (2016) 5:33–40. 27168953 PMC4858604

[52] Li F Zhang M Hussain F Triantaphyllopoulos K Clark AR Bhavsar PK. Inhibition of p38 MAPK-dependent bronchial contraction after ozone by corticosteroids. Eur Respir J. (2011) 37:933–42. 10.1183/09031936.00021110 20693246 PMC3331993

[53] Xu M Wang L Wang M Wang H Zhang H Chen Y. Mitochondrial ROS and NLRP3 inflammasome in acute ozone-induced murine model of airway inflammation and bronchial hyperresponsiveness. Free Radic Res. 53:780–90. 10.1080/10715762.2019.1630735 31185753

[54] Dinarello CA. Overview of the IL-1 family in innate inflammation and acquired immunity. Immunol Rev. (2018) 281:8–27. 10.1111/imr.12621 29247995 PMC5756628

[55] Michaudel C Maillet I Fauconnier L Quesniaux V Chung KF Wiegman C. Interleukin-1alpha mediates ozone-induced myeloid differentiation factor-88-dependent epithelial tissue injury and inflammation. Front Immunol. (2018) 9:916. 10.3389/fimmu.2018.00916 29867931 PMC5950844

[56] Gasse P Riteau N Charron S Girre S Fick L Petrilli V. Uric acid is a danger signal activating NALP3 inflammasome in lung injury inflammation and fibrosis. Am J Respir Crit Care Med. (2009) 179:903–13. 10.1164/rccm.200808-1274OC 19218193

[57] Franklin BS Mangan MS Latz E. Crystal formation in inflammation. Annu Rev Immunol. (2016) 34:173–202. 10.1146/annurev-immunol-041015-055539 26772211

[58] Michaudel C Mackowiak C Maillet I Fauconnier L Akdis CA Sokolowska M. Ozone exposure induces respiratory barrier biphasic injury and inflammation controlled by IL-33. J Allergy Clin Immunol. (2018) 142:942–58. 10.1016/j.jaci.2017.11.044 29331644

[59] Kim BG Lee PH Lee SH Park CS Jang AS. Impact of ozone on claudins and tight junctions in the lungs. Environ Toxicol. (2018) 33:798–806. 10.1002/tox.22566 29673049

[60] Kosmider B Loader JE Murphy RC Mason RJ. Apoptosis induced by ozone and oxysterols in human alveolar epithelial cells. Free Radic Biol Med. (2010) 48:1513–24. 10.1016/j.freeradbiomed.2010.02.032 20219673 PMC2965594

[61] Pulfer MK Taube C Gelfand E Murphy RC. Ozone exposure *in vivo* and formation of biologically active oxysterols in the lung. J Pharmacol Exp Ther. (2005) 312:256–64. 10.1124/jpet.104.073437 15316091

[62] McKinnon KP Madden MC Noah TL Devlin RB. *In vitro* ozone exposure increases release of arachidonic acid products from a human bronchial epithelial cell line. Toxicol Appl Pharmacol. (1993) 118:215–23. 10.1006/taap.1993.1027 8442000

[63] Wright DT Adler KB Akley NJ Dailey LA Friedman M. Ozone stimulates release of platelet activating factor and activates phospholipases in guinea pig tracheal epithelial cells in primary culture. Toxicol Appl Pharmacol. (1994) 127:27–36. 10.1006/taap.1994.1135 8048050

[64] Madden MCF Dailey LA Samet JM Inhibition of arachidonic acid esterification in human airway epithelial cells exposed to ozone *in vitro*. Inhal Toxicol. (1998) 10:795–811. 10.1080/089583798197466

[65] Sokolowska M Quesniaux VFJ Akdis CA Chung KF Ryffel B Togbe D. Acute respiratory barrier disruption by ozone exposure in mice. Front Immunol. (2019) 10:2169. 10.3389/fimmu.2019.02169 31608051 PMC6758598

[66] Michaudel C Fauconnier L Jule Y Ryffel B. Functional and morphological differences of the lung upon acute and chronic ozone exposure in mice. Sci Rep. (2018) 8:10611. 10.1038/s41598-018-28261-9 30006538 PMC6045627

[67] Pinart M Zhang M Li F Hussain F Zhu J Wiegman C. IL-17A modulates oxidant stress-induced airway hyperresponsiveness but not emphysema. PLoS ONE. (2013) 8:e58452. 10.1371/journal.pone.0058452 23505509 PMC3594315

[68] Sollberger G Tilley DO Zychlinsky A. Neutrophil extracellular traps: the biology of chromatin externalization. Dev Cell. (2018) 44:542–53. 10.1016/j.devcel.2018.01.019 29533770

[69] Benmerzoug S Ryffel B Togbe D Quesniaux VFJ. Self-DNA sensing in lung inflammatory diseases. Trends Immunol. (2019) 40:719–34. 10.1016/j.it.2019.06.001 31262653

[70] Benmerzoug S Rose S Bounab B Gosset D Duneau L Chenuet P. STING-dependent sensing of self-DNA drives silica-induced lung inflammation. Nat Commun. (2018) 9:5226. 10.1038/s41467-018-07425-1 30523277 PMC6283886

[71] Holze C Michaudel C Mackowiak C Haas DA Benda C Hubel P. Oxeiptosis, a ROS-induced caspase-independent apoptosis-like cell-death pathway. Nat Immunol. (2018) 19:130–40. 10.1038/s41590-017-0013-y 29255269 PMC5786482

[72] Scaturro P Pichlmair A. Oxeiptosis-a cell death pathway to mitigate damage caused by radicals. Cell Death Differ. (2018) 25:1191–3. 10.1038/s41418-018-0134-3 29844568 PMC6030169

[73] Scaturro P Pichlmair A. Oxeiptosis: a discreet way to respond to radicals. Curr Opin Immunol. (2019) 56:37–43. 10.1016/j.coi.2018.10.006 30342374

[74] Paludan SR Reinert LS Hornung V. DNA-stimulated cell death: implications for host defence, inflammatory diseases and cancer. Nat Rev Immunol. (2019) 19:141–53. 10.1038/s41577-018-0117-0 30644449 PMC7311199

[75] Bialas AJ Sitarek P Milkowska-Dymanowska J Piotrowski WJ Gorski P. The role of mitochondria and oxidative/antioxidative imbalance in pathobiology of chronic obstructive pulmonary disease. Oxid Med Cell Longev. (2016) 2016:7808576. 10.1155/2016/7808576 28105251 PMC5220474

[76] Prakash YS Pabelick CM Sieck GC. Mitochondrial dysfunction in airway disease. Chest. (2017) 152:618–26. 10.1016/j.chest.2017.03.020 28336486 PMC5812762

[77] Mustafa MG Cross CE. Effects of short-term ozone exposure on lung mitochondrial oxidative and energy metabolism. Arch Biochem Biophys. (1974) 162:585–94. 10.1016/0003-9861(74)90219-7 4366422

[78] Zychlinski L Raska-Emery P Balis JU Montgomery MR. Age-related difference in bioenergetics of lung and heart mitochondrial from rats exposed to ozone. J Biochem Toxicol. (1989) 4:251–4. 10.1002/jbt.2570040408 2634096

[79] Wiegman CH Michaeloudes C Haji G Narang P Clarke CJ Russell KE. Oxidative stress-induced mitochondrial dysfunction drives inflammation and airway smooth muscle remodeling in patients with chronic obstructive pulmonary disease. J Allergy Clin Immunol. (2015) 136:769–80. 10.1016/j.jaci.2015.01.046 25828268 PMC4559140

[80] Servais S Boussouar A Molnar A Douki T Pequignot JM Favier R. Age-related sensitivity to lung oxidative stress during ozone exposure. Free Radic Res. (2005) 39:305–16. 10.1080/10715760400011098 15788235

[81] Mustafa MG DeLucia AJ York GK Arth C Cross CE. Ozone interaction with rodent lung. II effects on oxygen consumption of mitochondria. J Lab Clin Med. (1973) 82:357–65. 4728288

[82] Li F Xu M Wang M Wang L Wang H Zhang H. Roles of mitochondrial ROS and NLRP3 inflammasome in multiple ozone-induced lung inflammation and emphysema. Respir Res. (2018) 19:230. 10.1186/s12931-018-0931-8 30466433 PMC6249848

[83] Li X Michaeloudes C Zhang Y Wiegman CH Adcock IM Lian Q. Mesenchymal stem cells alleviate oxidative stress-induced mitochondrial dysfunction in the airways. J Allergy Clin Immunol. (2018) 141:1634–45.e1635. 10.1016/j.jaci.2017.08.017 28911970

[84] Bao W Zhang Y Zhang M Bao A Fei X Zhang X. Effects of ozone repeated short exposures on the airway/lung inflammation, airway hyperresponsiveness and mucus production in a mouse model of ovalbumin-induced asthma. Biomed Pharmacother. (2018) 101:293–303. 10.1016/j.biopha.2018.02.079 29499403

[85] Kierstein S Krytska K Sharma S Amrani Y Salmon M Panettieri RA Jr. Ozone inhalation induces exacerbation of eosinophilic airway inflammation and hyperresponsiveness in allergen-sensitized mice. Allergy. (2008) 63:438–46. 10.1111/j.1398-9995.2007.01587.x 18315731

[86] Pichavant M Goya S Meyer EH Johnston RA Kim HY Matangkasombut P. Ozone exposure in a mouse model induces airway hyperreactivity that requires the presence of natural killer T cells and IL-17. J Exp Med. (2008) 205:385–93. 10.1084/jem.20071507 18250191 PMC2271004

[87] Lee LY Kwong K Lin YS Gu Q. Hypersensitivity of bronchopulmonary C-fibers induced by airway mucosal inflammation: cellular mechanisms. Pulm Pharmacol Ther. (2002) 15:199–204. 10.1006/pupt.2002.0338 12099764

[88] Bhalla DK. Ozone-induced lung inflammation and mucosal barrier disruption: toxicology, mechanisms, and implications. J Toxicol Environ Health B Crit Rev. (1999) 2:31–86. 10.1080/109374099281232 10081525

[89] Li F Wiegman C Seiffert JM Zhu J Clarke C Chang Y. Effects of N-acetylcysteine in ozone-induced chronic obstructive pulmonary disease model. PLoS ONE. (2013) 8:e80782. 10.1371/journal.pone.0080782 24260479 PMC3832609

[90] Mathews JA Kasahara DI Ribeiro L Wurmbrand AP Ninin FM Shore SA. γδ T cells are required for M2 macrophage polarization and resolution of ozone-induced pulmonary inflammation in mice. PLoS ONE. (2015) 10:e0131236. 10.1371/journal.pone.0131236 26135595 PMC4489797

[91] Salmon M Koto H Lynch OT Haddad EB Lamb NJ Quinlan GJ. Proliferation of airway epithelium after ozone exposure: effect of apocynin and dexamethasone. Am J Respir Crit Care Med. (1998) 157:970–7. 10.1164/ajrccm.157.3.9704067 9517619

[92] Zhang P Li F Wiegman CH Zhang M Hong Y Gong J. Inhibitory effect of hydrogen sulfide on ozone-induced airway inflammation, oxidative stress, and bronchial hyperresponsiveness. Am J Respir Cell Mol Biol. (2015) 52:129–37. 10.1165/rcmb.2013-0415OC 25010831

[93] Li F Zhang P Zhang M Liang L Sun X Li M. Hydrogen sulfide prevents and partially reverses ozone-induced features of lung inflammation and emphysema in mice. Am J Respir Cell Mol Biol. (2016) 55:72–81. 10.1165/rcmb.2015-0014OC 26731380

[94] Russell KE Chung KF Clarke CJ Durham AL Mallia P Footitt J. The MIF antagonist ISO-1 attenuates corticosteroid-insensitive inflammation and airways hyperresponsiveness in an ozone-induced model of COPD. PLoS ONE. (2016) 11:e0146102. 10.1371/journal.pone.0146102 26752192 PMC4709227

[95] Alexis NE Lay JC Haczku A Gong H Linn W Hazucha MJ. Fluticasone propionate protects against ozone-induced airway inflammation and modified immune cell activation markers in healthy volunteers. Environ Health Perspect. (2008) 116:799–805. 10.1289/ehp.10981 18560537 PMC2430237

[96] Chung KF Adcock IM. Induction of nuclear factor-kappa B by exposure to ozone and inhibition by glucocorticoids. Meth Enzymol. (2000) 319:551–62. 10.1016/S0076-6879(00)19052-4 10907543

[97] Haddad EB Salmon M Koto H Barnes PJ Adcock I Chung KF. Ozone induction of cytokine-induced neutrophil chemoattractant (CINC) and nuclear factor-kappa b in rat lung: inhibition by corticosteroids. FEBS Lett. (1996) 379:265–8. 10.1016/0014-5793(95)01524-8 8603703

[98] Haddad EB Liu SF Salmon M Robichaud A Barnes PJ Chung KF. Expression of inducible nitric oxide synthase mRNA in brown norway rats exposed to ozone: effect of dexamethasone. Eur J Pharmacol. (1995) 293:287–90. 10.1016/0926-6917(95)00032-1 8666049

[99] Haddad EB Salmon M Sun J Liu S Das A Adcock I. Dexamethasone inhibits ozone-induced gene expression of macrophage inflammatory protein-2 in rat lung. FEBS Lett. (1995) 363:285–8. 10.1016/0014-5793(95)00333-5 7737418

[100] Pinart M Hussain F Shirali S Li F Zhu J Clark AR. Role of mitogen-activated protein kinase phosphatase-1 in corticosteroid insensitivity of chronic oxidant lung injury. Eur J Pharmacol. (2014) 744:108–14. 10.1016/j.ejphar.2014.10.003 25310910 PMC4266539

[101] Adcock IM Marwick J Casolari P Contoli M Chung KF Kirkham P Mechanisms of corticosteroid resistance in severe asthma and chronic obstructive pulmonary disease (COPD). Curr Pharm Des. (2010) 16:3554–73. 10.2174/138161210793797889 20977420

[102] Chung KF Marwick JA Chung KF Adcock IM Molecular mechanisms of oxidative stress in airways and lungs with reference to asthma and chronic obstructive pulmonary disease multifaceted mechanisms in COPD: inflammation, immunity, and tissue repair and destruction. Ann N Y Acad Sci. (2010) 1203:85–91. 20716288 10.1111/j.1749-6632.2010.05600.x

[103] Chung KF. p38 mitogen-activated protein kinase pathways in asthma and COPD. Chest. (2011) 139:1470–9. 10.1378/chest.10-1914 21652557

[104] Perridon BW Leuvenink HG Hillebrands JL van Goor H Bos EM. The role of hydrogen sulfide in aging and age-related pathologies. Aging. (2016) 8:2264–89. 10.18632/aging.101026 27683311 PMC5115888

[105] Xu D Jin H Wen J Chen J Chen D Cai N. Hydrogen sulfide protects against endoplasmic reticulum stress and mitochondrial injury in nucleus pulposus cells and ameliorates intervertebral disc degeneration. Pharmacol Res. (2017) 117:357–69. 10.1016/j.phrs.2017.01.005 28087442

[106] Zheng J Zhao T Yuan Y Hu N Tang X. Hydrogen sulfide (H2S) attenuates uranium-induced acute nephrotoxicity through oxidative stress and inflammatory response via Nrf2-NF-κB pathways. Chem Biol Interact. (2015) 242:353–62. 10.1016/j.cbi.2015.10.021 26523793

[107] Chung KF. Hydrogen sulfide as a potential biomarker of asthma. Expert Rev Respir Med. (2014) 8:5–13. 10.1586/17476348.2014.856267 24308655

[108] Perry MM Tildy B Papi A Casolari P Caramori G Rempel KL. The anti-proliferative and anti-inflammatory response of COPD airway smooth muscle cells to hydrogen sulfide. Respir Res. (2018) 19:85. 10.1186/s12931-018-0788-x 29743070 PMC5944010

[109] Sun Y Wang K Li MX He W Chang JR Liao CC. Metabolic changes of H2S in smokers and patients of COPD which might involve in inflammation, oxidative stress and steroid sensitivity. Sci Rep. (2015) 5:14971. 10.1038/srep14971 26455818 PMC4601038

[110] Lin F Liao C Sun Y Zhang J Lu W Bai Y. Hydrogen sulfide inhibits cigarette smoke-induced endoplasmic reticulum stress and apoptosis in bronchial epithelial cells. Front Pharmacol. (2017) 8:675. 10.3389/fphar.2017.00675 29033840 PMC5625329

[111] Guan R Cai Z Wang J Ding M Li Z Xu J. Hydrogen sulfide attenuates mitochondrial dysfunction-induced cellular senescence and apoptosis in alveolar epithelial cells by upregulating sirtuin 1. Aging. (2019) 11:11844–64. 10.18632/aging.102454 31881011 PMC6949053

[112] Rogliani P Matera MG Page C Puxeddu E Cazzola M Calzetta L. Efficacy and safety profile of mucolytic/antioxidant agents in chronic obstructive pulmonary disease: a comparative analysis across erdosteine, carbocysteine, and N-acetylcysteine. Respir Res. (2019) 20:104. 10.1186/s12931-019-1078-y 31133026 PMC6537173

[113] Williams AS Issa R Leung SY Nath P Ferguson GD Bennett BL. Attenuation of ozone-induced airway inflammation and hyper-responsiveness by c-Jun NH2 terminal kinase inhibitor SP600125. JPharmacol ExpTher. (2007) 322:351–9. 10.1124/jpet.107.121624 17460151

[114] Williams AS Issa R Durham A Leung SY Kapoun A Medicherla S. Role of p38 mitogen-activated protein kinase in ozone-induced airway hyperresponsiveness and inflammation. EurJPharmacol. (2008) 600:117–22. 10.1016/j.ejphar.2008.09.031 18926814

[115] Hisada T Adcock IM Nasuhara Y Salmon M Huang TJ Barnes PJ. Inhibition of ozone-induced lung neutrophilia and nuclear factor-kappaB binding activity by vitamin A in rat. EurJPharmacol. (1999) 377:63–8. 10.1016/S0014-2999(99)00405-7 10448927

